# A Quick Measure of Theory of Mind in Autistic Adults: Decision Accuracy, Latency and Self-Awareness

**DOI:** 10.1007/s10803-021-05166-7

**Published:** 2021-06-28

**Authors:** Neil Brewer, Robyn L. Young, Jade Eloise Norris, Katie Maras, Zoe Michael, Emily Barnett

**Affiliations:** 1 College of Education, Psychology and Social Work, Flinders University, GPO Box 2100, Adelaide, 5001 Australia; 2 Bristol Medical School, University of Bristol, Bristol, UK; 3 Department of Psychology, University of Bath, Bath, UK

**Keywords:** Autism, Theory of Mind assessment, Decision latency, Metacognitive monitoring

## Abstract

Autistic adults often experience difficulties in taking the perspective of others, potentially undermining their social interactions. We evaluated a quick, forced-choice version of the Adult Theory of Mind (A-ToM) test, which was designed to assess such difficulties and comprehensively evaluated by Brewer et al. (2017). The forced-choice version (the A-ToM-Q) demonstrated discriminant, concurrent, convergent and divergent validity using samples of autistic (*N* = 96) and non-autistic adults (*N* = 75). It can be administered in a few minutes and machine-scored, involves minimal training and facilitates large-scale, live, or web-based testing. It permits measurement of response latency and self-awareness, with response characteristics on both measures enhancing understanding of the nature and extent of perspective taking difficulties in autistic individuals.

**Supplementary Information:**

The online version contains supplementary material available at 10.1007/s10803-021-05166-7.

Social interaction impairments that characterize autistic individuals are often attributed to difficulties in taking the perspective of others, difficulties which are thought to be reflective of Theory of Mind (ToM) limitations (Baron-Cohen, 1995; Baron-Cohen et al., 1985). There has been considerable debate about issues such as whether ToM is a core mechanism underpinning social difficulties in autistic individuals (e.g., Stone & Gerrans, 2006; Van de Cruys et al., 2014), whether other mechanisms (e.g., vocal or facial emotion processing, attention) are the key contributors (e.g., Globerson et al., 2015; Nuske et al., 2013), what the biological bases of autism-related difficulties in interpreting facial expressions may be (e.g., Critchley et al., 2000), or whether autistic individuals are uniquely or even universally impaired on ToM tasks (Gernsbacher & Yergeau, 2019). It is important to note that a host of other issues that are clearly relevant to a comprehensive understanding of ToM difficulties in autistic individuals have been canvassed, especially by those interested in more basic research questions about ToM. For example, such researchers have distinguished between constructs such as cognitive (i.e., thinking about the thoughts and beliefs of others) and affective (i.e., thinking about their feelings or emotions) ToM (e.g., Dvash & Shamay-Tsoory, 2014), and between two distinct but complementary ToM systems that, respectively, underpin efficient and relatively automatic mental state inferences, and more flexible but effortful reasoning about mental states (Apperly & Butterfill, 2009).

Our focus is, however, on the following issue. On tests where adult participants have to decode the meaning of social interactions captured on video, autistic individuals—at least at the group level—tend to perform less effectively than non-autistic adults (e.g., Brewer et al., 2017; Dziobek et al., 2006; Heavey et al., 2000). Instruments that measure such perspective taking difficulties provide researchers with a tool for understanding factors that may underlie problematic social interactions, and for developing guidelines to improve social-communicative skills. For clinicians, use of such measures allows them to pinpoint specific difficulties that individual clients might experience when trying to understand other people’s social communications, and to demonstrate to clients some of the subtleties of interpersonal interactions that they may be missing. Moreover, such formal measures provide the added benefit of being able to confirm—as highlighted by the heterogeneity in perspective taking abilities of autistic adults demonstrated by Brewer et al. (2017)—that some adults are unlikely to be experiencing difficulties in these areas.

Yet, many existing instruments are cumbersome in terms of administration, scoring, and the training thereof, thereby limiting their usefulness. Here we demonstrate that by substituting a forced-choice response format for the free-report format of the Adult Theory of Mind (A-ToM) test (Brewer et al., 2017)—a test subjected to a comprehensive psychometric evaluation with a large sample of autistic adults—an efficient measure from training, administration, and scoring perspectives is created. This forced-choice measure enables recording of response latency, confidence and decision accuracy, providing a nuanced picture of perspective taking difficulties.

## Measuring Perspective Taking in Adults with ASD

Numerous measures designed to identify adults’ mental state inferences when observing people interacting in realistic everyday social situations have been developed (e.g., Brewer et al., 2017; Dziobek et al., 2006; Heavey et al., 2000). They have primarily addressed the nature of autism-related difficulties, with the associated measurement foci and the resulting sample sizes not geared towards formal psychometric evaluations. The Adult Theory of Mind or A-ToM (Brewer et al., 2017)—an adaptation and substantial extension of the Strange Stories test (Happé, 1999)—was, however, designed as a formal assessment tool. Its evaluation targeted sample sizes that permitted a systematic psychometric evaluation, encompassing item analysis, reliability assessment, and examinations of the instrument’s factor structure, concurrent, divergent and discriminant validity. That evaluation identified six social, or perspective taking, items (e.g., interpreting elements such as sarcasm, faux pas, white lie, bluff, and misunderstanding) that differentiated autistic and non-autistic individuals, and six physical or control items that were less sensitive to autistic characteristics, with the required cognitions not involving social or perspective taking inferences).

Brewer et al. (2017) showed that the A-ToM was characterized by sound inter-rater and test–retest reliability. Its factor structure reflected social and physical dimensions, with autistic and non-autistic participants more strongly differentiated on the social than the physical scale. Unsurprisingly, given the heterogeneous nature of autism, and despite the significant group differences on the A-ToM’s social scale, inter-individual variability was marked, with some overlap between the performance of autistic and non-autistic individuals. Concurrent validity was evidenced by correlations with the corresponding sub-scales of two widely used ToM measures: the Strange Stories test (Happé, 1999) and the Frith-Happé animations (White et al., 2011). Subsequently, researchers have demonstrated criterion-related validity through correlations of autistic adults’ A-ToM social performance with measures of their social–behavioral skills and interpersonal relationships (Brewer et al., 2019), and with their ability to extricate themselves from situations in which they were erroneously suspected of involvement in a crime (Young & Brewer, 2020), thereby highlighting the likely critical contributions of perspective taking difficulties to adaptive social functioning. Divergent validity was illustrated by correlations with the perspective taking and empathic concern sub-scales of the Interpersonal Reactivity Index’s (IRI; Davis, 1983), but not the IRI’s personal distress sub-scale or a measure of social anxiety, the mini-SPIN (Connor et al., 2001), despite the latter two measures clearly differentiating autistic and non-autistic individuals.

Although the A-ToM is a promising measure for researchers and clinicians exploring the limitations imposed by adults’ perspective taking difficulties in day-to-day social functioning, it lacks some features that would make it more useful. As Livingston et al. (2019) argued, key issues for users are training requirements and administration time, scoring complexity and time, and the potential for web-based administration and automated scoring, factors that would make assessment more accessible and facilitate large-scale data collection. Administration time for the A-ToM is 15–20 min. Administrators require training and practice to ensure reliable coding of responses. Training of each coder takes approximately 1 h, and coding of all items by experienced personnel takes 15–20 min. Because A-ToM responses are provided by free report, measures of response latency are confounded by individual differences in factors such as motor proficiency and expressive ability. Yet, as Livingston et al. (2019) highlighted, response latency is potentially informative, not only about processing difficulties but also about responsiveness to clinical interventions. In many live interpersonal interactions, individuals often do not have the opportunity to reflect (at least for very long) on the meaning of or intentions behind another individual’s verbal and nonverbal communications (cf. Apperly & Butterfill, 2009). Thus, the speed with which an individual grasps the perspective of others and responds appropriately will often be the key to adaptive and appropriate social interactions.

The current study was designed to overcome the limitations of the A-ToM, without modifying test content. We substituted a forced-choice response format for the free-report of the A-ToM, thereby producing the quick form of the A-ToM, labeled the A-ToM-Q. Each item was accompanied by four multiple-choice alternative responses (one of which was the correct answer), with these alternatives taken directly from the A-ToM’s coding protocols as described in Brewer et al. (2017). To obtain a response latency measure, participants received a standard processing speed task instruction: respond as quickly and as accurately as possible.

We also examined participants’ self-awareness of their perspective taking strengths and limitations by obtaining a confidence measure for each response. Being aware of one’s capabilities in reading the intentions or perspective of others—referred to as metacognitive monitoring—should increase the likelihood that the individual will respond in a socially appropriate manner during interpersonal interactions and allow them to identify possible areas for improvement (Kelly & Metcalfe, 2011). Self-awareness is indicated if the individual’s confidence and accuracy in their perspective taking responses are meaningfully related. A common approach to assessing this relationship is to calculate a point-biserial correlation, with confidence (expressed on a scale such as 0%, 10%, …, 100%) correlated with a binary accuracy outcome (correct, incorrect). Although the point-biserial correlation reveals how well confidence discriminates correct and incorrect decisions, it is now well established that a weak point-biserial correlation may conceal meaningful relationships (for detail, see Brewer & Wells, 2006; Juslin et al., 1996). A more informative assessment of the relationship involves using a calibration approach which charts the relationship between subjective and objective probabilities of accuracy by systematically plotting accuracy against each level of confidence (e.g., Brewer & Wells, 2006; Juslin et al., 1996). If an individual’s confidence and accuracy in their perspective taking responses are well calibrated, accuracy should increase systematically with increases in confidence. Thus, perfect calibration is evident if the obtained calibration curve is linear, with all decisions made with 100% confidence being accurate, 90% of responses made with 90% confidence accurate, and so on. In other words, the calibration approach indicates the likely accuracy of decisions made with particular levels of confidence, and can speak to the individual’s awareness of the accuracy of their own perspective taking judgments, thus indicating whether their judgments are characterized by over- or underconfidence.

We expected autistic and non-autistic individuals to be more strongly differentiated on the A-ToM-Q’s social (i.e., perspective taking) scale than its physical scale. We also expected significant correlations between the social sub-scale and extant ToM scales, including the Strange Stories tests’ social scale (Happé, 1999) and the feelings categorization and mental scales of the Frith-Happé animations (White et al., 2011). Although we expected autistic and non-autistic participants to differ on self-report measures of perspective taking, empathic concern, personal distress and social anxiety, we only expected the perspective taking and empathic concern measures to correlate with the A-ToM-Q social sub-scale.

Latency patterns were difficult to predict given we were unable to control individuals’ speed-accuracy operating characteristics, despite instructions emphasizing both speed and accuracy. One possibility is suggested by research showing that, unlike non-autistic individuals who show a predisposition towards an intuitive style of decision making, autistic individuals and individuals with more autistic traits may be predisposed towards deliberative or effortful processing (e.g., Brosnan et al., 2016b, 2017). Consistent with this view is Miu et al. (2012) finding of longer response times on the Reading the Mind in the Eyes Test (Baron-Cohen et al., 2001a) for (neurotypical) individuals high on autistic traits (cf. those with low autistic traits) as measured by the Autism Spectrum Quotient (AQ; Baron-Cohen et al., 2001b). Under forced choice responding such response patterns would lead to longer response latencies for the ASD group, possibly regardless of any group differences in accuracy. Another logical, though unlikely, possibility is that poorer social scale performance by autistic individuals would reflect careless responding, with autistic individuals responding as rapidly as or even faster than non-autistic individuals.

Possible group differences in self-awareness were also difficult to forecast. Investigations of metacognitive monitoring in autistic and non-autistic samples in several different task domains have been reported, with mixed findings. For example, poorer metacognitive monitoring by autistic individuals has been reported on general knowledge and mathematics tasks (Brosnan et al., 2016a; Grainger et al., 2016) but not on an episodic memory task (Maras et al., 2020). On a facial emotion recognition task, perhaps the closest approximation to a perspective taking task, Sawyer et al. (2014) reported comparable discrimination between confidence for correct and incorrect responses for autistic and non-autistic samples. However, contrasting average confidence for correct and incorrect responses only taps one aspect of the confidence-accuracy relationship and provides no guide as to the sensitivity of individuals’ adjustments in confidence in response to the range of possible variations in accuracy. The latter information is provided by the confidence-accuracy calibration approach that has been widely used in different decision-making domains (e.g., Baranski & Petrusic, 1994; Brewer & Wells, 2006; Maras et al., 2020): the essence of this approach is to track accuracy variations across the range of possible confidence judgments.

Here, autistic and non-autistic adults completed the A-ToM-Q, with the key dependent measures being response accuracy, latency, and confidence for social and physical items. Relationships with existing ToM scales (the Strange Stories and Frith-Happé animations) indexed concurrent validity. Other measures likely to reflect ToM (the IRI’s perspective taking and empathic concern scales) were used to assess convergent validity, while two measures (the IRI’s personal distress scale and the mini-SPIN social anxiety scale) expected to differentiate the two samples, independent of ToM, were used to assess divergent validity.

## Method

### Participants

The autistic sample was recruited from two sources with the aim of securing approximately 100 adults with verbal abilities likely to exclude the possibility of an intellectual disability. Thirty-two adults (12 female) diagnosed with Asperger syndrome (AS) or ASD and registered on an Australian university research participation database comprised one source. They were aged from 20 to 64 years (*M* = 33.4, SD = 14.0). Verbal Comprehension Index (VCI) and Full-Scale IQ (FSIQ) scores on the Wechsler Abbreviated Scale of Intelligence-Second Edition (WASI-II; Wechsler, 2011) ranged from 85 to 149 (*M* = 104.3, *SD* = 13.7, 95% *CI* [99.5, 109.2]) and 85–146 (*M* = 105.5, *SD* = 14.1, 95% *CI* [100.6, 110.5], respectively. Participants met DSM-IV-TR [American Psychiatric Association, 2000] or DSM-5 [American Psychiatric Association, 2013] criteria, and had been diagnosed by two qualified diagnosticians or a psychologist endorsed by the local autism service delivery agency. One individual completed all measures but was excluded because their WASI-II VCI was below 85. Participants received an honorarium for participation.

Another 74 individuals were recruited via a UK university autism research database and social media and completed all measures using the Qualtrics platform. Autism diagnoses were ascertained by asking participants to provide detailed information about their diagnosis, including confirmation of a formal diagnosis by a qualified clinical professional, and details regarding the diagnosis type (e.g., autism, ASD, ASC, Asperger Syndrome, etc.), age at diagnosis, diagnosis date and location, and the diagnostician. WASI-II administration was not possible using the online delivery. Instead, verbal comprehension was measured using Part 1 of the Advanced Vocabulary Test I-V-4 (AVT) from Educational Testing Services’ Kit of Factor-Referenced Cognitive Tests (Ekstrom et al., 1976). Participants (*N* = 3) scoring more than two standard deviations below the normative sample mean were excluded. A detailed comparison of all dependent measures obtained from the two ASD sub-samples is presented in the Results section. Six other individuals completed the assessments but were excluded due to audio failures during A-ToM-Q presentation or because the participant participated twice. The final online sample comprised 65 individuals (43 female) aged 18 to 60 years (*M* = 35.5, *SD* = 12.3). AVT scores ranged from 4.25 to 18 (*M* = 10.4, *SD* = 3.3, 95% *CI* [9.59, 11.2]). The AQ-10 (Autism Spectrum Quotient; Allison et al., 2012) profile of the online sample was consistent with the presence of autistic characteristics (*M* = 7.97, *SD* = 1.98, 95% *CI* [7.49, 8.45]). Thus, overall, the autistic sample comprised 96 individuals, aged 18 to 64 years (*M* = 34.8, *SD* = 12.9).

The non-autistic sample comprised 81 individuals, mostly students enrolled in undergraduate programs or programs designed to facilitate transition to university study for mature-aged students. Five individuals were excluded because their WASI-II VCI was less than 85; and one individual was excluded for scoring 6 or higher on the AQ-10, suggesting grounds for a diagnostic assessment for ASD (Allison et al., 2012). The final sample included 75 non-autistic individuals (56 female) whose ages ranged from 17 to 50 years (*M* = 22.4, *SD* = 6.8). WASI-II VCI and FSIQ scores ranged from 86 to 126 (*M* = 104.9, *SD* = 9.0, 95% *CI* [102.8, 107.0]) and 83–130 (*M* = 105.4, *SD* = 10.8, 95% *CI* [102.9, 107.8]), respectively. Their AQ-10 scores were markedly lower than those of the online ASD sub-sample, (*M* = 2.40, *SD* = 1.63, 95% *CI* [2.03, 2.78] vs. *M* = 7.97, *SD* = 1.98, 95% *CI* [7.49, 8.45]).

### Materials

*A-ToM-Q*. The A-ToM-Q used the six social and six physical stimulus items from the final scale of the A-ToM (Brewer et al., 2017). Each scenario was scripted, acted, and filmed to produce a professional quality set of digital video stimuli. The scenarios ranged in duration from 14 to 108 s. (﻿Copies of the stimuli may be viewed at the URLs below. Two examples of social item video scripts are presented in the Appendix. Bona fide researchers and clinicians will be able to access the stimuli free-of-charge from a site managed by the researchers.)

Social Playlist Link:

https://www.youtube.com/playlist?list=PLJCW1evzKKctzHvYfB1RADd27m8IBaWcu

Physical Playlist Link:

https://www.youtube.com/playlist?list=PLJCW1evzKKcuy1rGu3Ocatm97s_KpdhyI

The order of item presentation was randomized. Each item was followed by four forced-choice alternative response options; the four alternatives for each question were randomly ordered and the resulting order was used for all participants. Participants were instructed to select one of the forced-choice options as quickly and accurately as possible.

We used specific examples from the original A-ToM’s scoring protocols (Brewer et al., 2017) to produce the four response alternatives for each item: one alternative matched the correct response, one or two alternatives matched responses coded as partially correct, and one or two matched incorrect responses. The partially correct response options were included among the four alternatives to ensure challenging discriminations, not for scoring purposes (see below). Whether one or two partially correct or incorrect alternatives were used for each item was based on the number of clear exemplars in the A-ToM’s coding guidelines that could be translated directly into a multiple-choice response option. The alternatives and scoring protocols for all items appear in Supplemental Materials Table S1.

Answers were scored 0 (incorrect) or 1 (correct), with possible scores on each sub-scale ranging from 0 to 6. The A-ToM’s scoring protocol (Brewer et al., 2017) was 0 (incorrect), 1 (partially correct) and 2 (correct). However, with only four alternatives per item for the A-ToM-Q’s forced-choice version, assigning points to partially correct alternatives for each item meant that participants could score a point by selecting any of 2 or, for some items, 3 of the response options, thereby potentially inflating accuracy scores by chance. Accordingly, although partially correct alternatives were retained in the forced-choice options to ensure challenging discriminations, responses scored partially correct using the original A-ToM’s scoring protocols received 0 on the multiple-choice version. Item-total correlations ranged from 0.29 to 0.46 for social items and 0.00 to 0.27 for physical items (coefficient alpha is not reported due to the widely reported concerns about its interpretation; e.g., McNeish, 2018).

Response latency for each item was recorded from the appearance of the multiple-choice alternatives to the participant’s mouse click on their chosen alternative. Following each response, participants rated their confidence in their answer from 0 (absolutely uncertain) to 100% (absolutely certain), on an 11-point decile scale.

#### Verbal Ability

The WASI-II comprises Vocabulary, Similarities, Block Design, and Matrix Reasoning subtests, with the Vocabulary and Similarities subtests making up the Verbal Comprehension Index (VCI). The Advanced Vocabulary Test I-V-4 (AVT) from Educational Testing Services’ Kit of Factor-Referenced Cognitive Tests (Ekstrom et al., 1976) comprises 18 items that assess knowledge of word meanings, completed within a 4-min time limit. On each item, participants are presented with a word and asked to select its synonym from five options. Participants’ scores on the test are the number of items answered correctly minus a fraction of the number of items answered incorrectly (participants may opt not to answer items if they are uncertain). Scores on this measure can therefore range from − 4.5 to 18, with higher scores indicating higher levels of verbal comprehension.

#### Concurrent Validity Measures

The measures were the Strange Stories test (Happé, 1999) and the Frith-Happé animations (White et al., 2011). The Strange Stories test comprised 16 scenarios or stories (8 social, 8 physical) for which the examinee provides a non-literal, free-report interpretation of the meaning of scenario characters’ expressions. The second concurrent validity measure was White et al.’s (2011) modification of Frith and Happé’s (Abell et al., 2000) animations of two triangles moving around on the screen either randomly or apparently in some kind of response to each other. Participants viewed 14 videos in total: 2 (practice trial) videos, 4 ToM (i.e., social or mental) videos, 4 goal-directed (physical) videos, and 4 random videos. After each video participants were asked to indicate whether the behavior displayed by the triangles involved a mental (i.e., social) interaction, a purely physical interaction or no interaction. Whenever they correctly identified a mental interaction, they then selected the word from a list that best described how each of the triangles were feeling at the end of the video. These responses gave rise to a feelings categorization score. White et al. (2011) reported that, compared with non-autistic controls, autistic adults performed more poorly at recognizing mental state interactions and had lower feelings categorization scores.

#### Convergent and Divergent Validity Measures

Two convergent measures were provided by self-report social-behavioral skills (18 items) and interpersonal relationships’ (13 items) questionnaires, reflecting varied aspects of the individual’s adult relationships. In a sample of autistic individuals, Brewer et al. (2019) reported that ToM (measured by the A-ToM) was significantly related to both measures (*r* = 0.35 and 0.64).

Other measures were provided by three sub-scales of the IRI (Davis, 1983). Each scale comprises seven items probing the extent to which individuals reported (a) taking the psychological perspective of others, (b) showing empathic concern for others in difficulty, and (c) feeling personal distress in tense interpersonal settings. The final measure was the Mini-SPIN, a three item, self-report screener for generalized social anxiety disorder (Connor et al., 2001). All measures were expected to distinguish autistic and non-autistic individuals, but significant correlations were not expected between the A-ToM-Q social scale and the divergent measures of personal distress and anxiety disorder.

### Procedure

Participants received details of what was required, read a study information sheet, and gave informed consent. Online participants confirmed that they were using a laptop device or similar, answered two questions designed to exclude bots, and responded to screening questions ensuring normal or corrected to normal vision and hearing, and no history of major psychiatric, or neurocognitive disorders. They then provided details of their age, gender, native language, and ASD diagnosis. For all participants, tests were administered in the following order: A-ToM-Q (item order random), AQ-10, Mini-SPIN, IRI, AVT (or WASI-II), Social and Behavioral Skills, Interpersonal Skills, Frith-Happé animations, and Strange Stories (physical and social items counterbalanced). We administered tests in this order (rather than counterbalancing) to ensure optimal attention for the key A-ToM-Q measure, which was separated as much as possible from the other ToM measure (i.e., the Strange Stories test). Testing took place at the university or online (UK ASD sub-sample). Participants were told to expect the tasks to take at least two hours to complete and they could take breaks when needed during the session. The study received ethical approval from the appropriate ethics review committee at each institution.

## Results

### Comparison of Autistic Sub-Samples

We compared the UK and Australian sub-samples of autistic individuals on all measures obtained from both samples to alleviate concerns about combining them. Significant group differences were only detected on the IRI’s self-report perspective taking scale, *t* (94) = 2.28, *p* = 0.025, *d* = 0.50, 95% *CI* [0.06, 0.93], and the Mini-Spin, *t* (94) = 2.09, *p* = 0.039, *d* = 0.46, 95% *CI* [0.03, 0.89]; the UK sample scored lower on perspective taking and higher on social anxiety than the Australian sample. As the sub-samples did not differ significantly on the A-ToM-Q, the Strange Stories test, the Frith-Happé animations, or the convergent and divergent validity measures, the two sub-samples were combined.

### Validity

#### Discriminant Validity

A-ToM-Q social sub-scale performance was significantly lower for the autistic (*M* = 4.24, *SD* = 1.65, 95% *CI* [3.91, 4.57]) than the non-autistic sample (*M* = 5.27, *SD* = 0.81, 95% *CI* [5.09, 5.45]), *t* (144.91) = 5.23, *p* < 0.001, *d* = 0.76, *CI* [0.45, 1.07]. A-ToM-Q physical performance was also significantly lower for the autistic (*M* = 3.64, *SD* = 1.33, 95% *CI* [3.37, 3.91]) than the non-autistic sample (*M* = 4.25, *SD* = 1.21, 95% *CI* [3.98, 4.52]), *t* (169) = 3.14, *p* = 0.002, *d* = 0.47, *CI* [0.16, 0.78].^1^ However, the magnitude of the difference between the two groups for the social scale was larger, with the effect size index approaching large for the social measure compared with weak-moderate for the physical measure.

It is unlikely that the A-ToM-Q social group difference was IQ-related. For the autistic and non-autistic individuals tested with the WASI-II, correlations between the social score and VCI (*r* = 0.07) and FSIQ (*r* = 0.01) were negligible. In contrast, significant moderate correlations were detected between the physical score and both VCI (*r* = 0.29) and FSIQ (*r* = 0.32). A one-way ANCOVA on the A-ToM-Q social scores for the autistic and non-autistic sub-groups, with VCI as the covariate, confirmed the significant effect of group, *F* (1, 103) = 9.94, *p* = 0.002, *η*_*p*_^2^ = 0.09. In contrast, an identical analysis on the A-ToM-Q physical scores revealed that the significant effect of group disappeared after controlling for VCI, *F* (1, 103) = 2.53, *p* = 0.12, *η*_*p*_^2^ = 0.02. For those autistic individuals who completed the AVT verbal ability measure, correlations between the A-ToM-Q social and physical scores and the AVT were also weak (*r* = 0.08 and 0.13, respectively) and non-significant. Although not all autistic participants completed the WASI-II, these data patterns indicate that not only did the A-ToM-Q social scale differentiate the two groups more clearly than the physical scale, but also that performance on the social (though not the physical) scale was independent of verbal ability.

#### Concurrent Validity

As expected, significant positive correlations were detected between (a) the A-ToM-Q social scale and both the Strange Stories social sub-scale (*r* = 0.45, *p* < 0.01) and the Frith-Happé animations’ mental and feelings categorization scales (*r*s = 0.17, *p* < 0.05, and 0.28, *p* < 0.01. respectively), and (b) the A-ToM-Q physical scale and the Strange Stories physical scale (*r* = 0.37, *p* < 0.01). Supplemental Materials Table S2 provides the complete inter-correlation matrix. Descriptive statistics for the two groups on the Strange Stories and Frith-Happé animations sub-scales are provided in Supplemental Materials Table S3. For the Strange Stories, the effect size indices reveal that the differentiation between autistic and non-autistic adults was more pronounced for physical than social items, the opposite of the expected pattern. The two groups were significantly differentiated on the Frith-Happé animations’ mental and feelings categorization measures, but with relatively weak effect sizes, similar to those reported by Brewer et al. (2017) for the A-ToM.

#### Convergent and Divergent Validity

As expected, autistic participants scored more poorly than non-autistic participants on both the social-behavioral skills and the interpersonal relationships measure (see Supplemental Materials Table S3), with both measures correlating significantly with the A-ToM-Q social score, *r* = 0.35 and 0.30, *p*s < 0.01, respectively. The A-ToM-Q social score also correlated significantly with the IRI’s perspective taking, *r* = 0.27, and empathic concern sub-scales, *r* = 0.25, *p*s < 0.01, although the autistic sample only differed significantly from the non-autistic sample on the former sub-scale (see Supplemental Materials Table S3). Finally, evidence for divergent validity was provided by the findings that although the autistic sample scored markedly higher than the non-autistic sample on both the IRI personal distress sub-scale and the Mini-SPIN social anxiety scale (see Supplemental Materials Table S3), neither measure correlated meaningfully with the A-ToM-Q social sub-scale, *r*s = − 0.01 and − 0.15, respectively, *p*s > 0.05.

### Decision Latency

Our presentation of the decision latency data was guided by several objectives. One was to provide detailed descriptive data by test item for each group to provide something akin to a preliminary normative reference base to assist interpretation of test responding. A second objective was to explore group differences in speed-accuracy operating characteristics. For example, when the decision latency and social scale accuracy data are considered together, might they point to group differences in (a) the tendency to favor deliberative or intuitive processing, reflected in decision latency differences regardless of accuracy, (b) response caution, perhaps with speed of responding sacrificed for accuracy (or vice versa) (c) the time needed to achieve equivalent levels of accuracy, or (d) perhaps a combination of some fundamental limitation affecting accuracy and a tendency to favor either deliberative or intuitive processing? A third objective was to compute a composite latency measure that facilitated examination of the relationship between ToM and decision latency.

Given the unsurprising inter-item variations in both latency and the number of correct and incorrect responses, our primary focus was a descriptive examination of patterns that were consistent across items. Descriptive latency statistics for each group are presented in Table 1. Any outliers (identified using *z* =  ± 3.29) were first assigned the next most extreme value ± 1 unit of measurement (0.01 s). Three noteworthy patterns emerged. First, as shown in Table 1, both groups’ latencies for correct decisions were generally shorter than those for incorrect decisions, a pattern that response latency researchers have argued indicates that incorrect decisions more likely reflect difficult discriminations than careless responding (cf. Brewer & Smith, 1984, 1989). Second, for correct decisions, latencies for the autistic sample were significantly longer than those for non-autistic individuals, with the exception of just one physical item (lightbulb) for which the difference was much smaller (see Table 2 for inferential contrasts and associated effect sizes for each item). Third, for incorrect decisions, the effect size indices reveal that the group differences in latency were less pronounced than for correct decisions for 6 (3 social, 3 physical) of the 12 items (see Table 2) although, for 5 of the 12 items, incorrect response latencies for non-autistic individuals are based on too few responses (e.g., 1–10) to provide stable estimates.

**Table 1:** Decision latency descriptive statistics (in sec) for autistic and non-autistic groups on A-ToM-Q social and physical items

A-ToM-Q Item	Autistic			Non-autistic		
	Mean	SD	Median	Mean	SD	Median
Social						
Bunnies						
Correct	12.37	8.98	9.53	6.18	3.69	5.05
Incorrect	16.48	18.28	12.30	13.56	5.16	13.36
Party						
Correct	19.78	9.88	17.29	12.86	10.00	9.77
Incorrect	22.93	9.49	19.25	13.83	-	13.83
Crying man						
Correct	9.47	6.06	7.65	5.24	5.20	3.44
Incorrect	15.89	9.10	3.44	21.64	10.35	21.64
Burglar						
Correct	13.06	8.40	10.36	7.22	5.40	5.36
Incorrect	14.09	7.97	12.57	12.21	9.61	9.11
Hat						
Correct	9.23	4.84	7.67	6.24	3.57	5.36
Incorrect	9.03	4.90	7.41	7.79	5.36	6.83
Spaghetti						
Correct	12.10	7.25	10.47	6.91	4.99	5.43
Incorrect	15.79	9.29	11.51	8.76	4.35	7.54
Physical						
Lightbulb						
Correct	9.12	6.34	7.11	7.17	4.47	6.04
Incorrect	12.24	7.77	10.25	7.23	6.86	5.60
Swimming						
Correct	7.14	3.81	6.02	4.95	2.71	4.37
Incorrect	11.93	5.51	10.45	8.85	7.57	4.89
Glasses						
Correct	11.62	6.34	10.96	8.63	4.65	7.32
Incorrect	15.61	7.63	12.29	12.14	8.45	8.31
Car						
Correct	12.11	8.08	9.42	7.10	4.39	5.71
Incorrect	12.90	6.54	11.26	12.08	7.34	9.48
Leg injury						
Correct	10.39	5.56	8.62	6.80	3.50	6.22
Incorrect	9.50	6.33	6.66	9.32	3.42	10.86
Librarian						
Correct	10.27	4.96	9.93	6.50	3.45	6.30
Incorrect	12.88	6.22	11.31	10.96	3.54	10.76

**Table 2:** Decision latency inferential statistics (in sec) for autistic versus non-autistic group contrasts on A-ToM-Q social and physical items

Item/response	Statistic		
	*t*	Cohen’s *d*	95% CI around *d*
Social			
Bunnies			
Correct	*t* (94.67) = − 5.33, *p* < .001	0.89	0.54, 1.24
Incorrect	*t* (33) = − .20, *p* = .846	0.07	− 0.66, 0.80
Party			
Correct	*t* (152) = − 4.31, *p* < .001	0.70	0.37, 1.02
Incorrect	*t* (15) = − .93, *p* = .367	0.96	− 1.10, 2.99
Crying man			
Correct	*t* (142.29) = -4.54, *p* < .001	0.75	0.41, 1.08
Incorrect	*t* (22) = .85, *p* = .405	0.63	− 0.84, 2.08
Burglar			
Correct	*t* (101.51) = − 4.49, *p* < .001	0.82	0.44, 1.20
Incorrect	*t* (53) = − .77, *p* = .444	0.22	− 0.34, 0.78
Hat			
Correct	*t* (102.62) = − 3.80, *p* < .001	0.71	0.34, 1.08
Incorrect	*t* (51) = − .79, *p* = .432	0.25	− -0.36, 0.86
Spaghetti			
Correct	*t* (113.42) = − 4.77, *p* < .001	0.84	0.48, 1.20
Incorrect	*t* (29.44) = − 3.18, *p* = .003	0.83	0.06, 1.59
Physical			
Lightbulb			
Correct	*t* (93) = − 1.75, *p* = .084	0.36	− 0.05, 0.77
Incorrect	*t* (74) = − 2.74, *p* = .008	0.67	0.18, 1.16
Swimming			
Correct	*t* (146.62) = − 4.14, *p* < .001	0.65	0.32, 0.98
Incorrect	*t* (17) = − 1.01, *p* = .327	0.50	− 0.49, 1.47
Glasses			
Correct	*t* (102) = − 2.77, *p* = .007	0.55	0.15, 0.94
Incorrect	*t* (65) = − 1.58, *p* = .119	0.44	− 0.12, 0.99
Car			
Correct	*t* (83.05) = − 3.86, *p* < .001	0.75	0.33, 1.16
Incorrect	*t* (73) = − .51, *p* = .613	0.12	− 0.34, 0.58
Leg injury			
Correct	*t* (109.29) = − 4.32, *p* < .001	0.76	0.39, 1.13
Incorrect	*t* (46.84) = − .13, *p* = .896	0.03	− 0.55, 0.61
Librarian			
Correct	*t* (97) = − 4.23, *p* < .001	0.86	0.44, 1.27
Incorrect	*t* (61.87) = − 1.64, *p* = .105	0.37	− 0.10, 0.84

*negative *t* statistic indicates longer latency for autistic than for non-autistic group

Given the different age ranges and mean age for the two groups, and the likelihood of age effects on decision latency (cf. Smith & Brewer, 1995), we examined the relationship between age and decision latency for correct A-ToM-Q social responses using a composite latency measure for each participant. As decision latency varied markedly across scenarios, we first translated each individual’s latency for each item into a standard score on that item. Next, to check whether individuals’ response latencies across items were reasonably consistent—for example, fast (slow) individuals were generally fast (slow) across items—we examined the inter-correlations between the combined autistic and non-autistic samples’ standard scores for each item. All correlations between the A-ToM-Q social items were statistically significant and mostly in the 0.4–0.5 range (see Supplemental Materials Table S4). Accordingly, we computed an average z-score for each participant to use as a composite latency measure for correct responses. Decision latency was positively correlated with age in both the autistic group, *r* = 0.27, *p* =  < 0.01, and the non-autistic group, *r* = 0.28, *p* = 0.02. We then re-examined decision latency differences between the groups. Table 3 shows the adjusted mean latencies for the social items—the items of primary interest—for the two groups, and the inferential contrasts of latencies for the two groups with age controlled. Although the group latency differences remained across items, the effect sizes were reduced.

**Table 3:** Decision latency descriptive statistics (in sec) and one-way ANCOVA inferential statistics, with age as a covariate, for autistic and non-autistic groups’ correct decisions on A-ToM-Q social and physical items

A-ToM-Q item	Autistic		Non-autistic	
	Adjusted mean	SE	Adjusted mean	SE
Social				
Bunnies	11.28	0.88	7.49	0.92
	*F* (1, 132) = 7.61, *p* = .007, *d* = 0.48			
Party	18.12	1.15	14.25	1.20
	*F* (1, 150) = 4.74, *p* = .031, *d* = 0.36			
Crying man	8.34	0.66	6.38	0.67
	*F* (1, 144) = 3.83, *p* = .052, *d* = 0.33			
Burglar	11.95	1.00	8.21	1.03
	*F* (1, 112) = 5.78, *p* = .018, *d* = 0.45			
Hat	9.01	0.61	6.44	0.59
	*F* (1, 115) = 7.87, *p* = .006, *d* = 0.52			
Spaghetti	12.17	0.83	6.71	0.82
	*F* (1, 127) = 19.27, *p* < .001, *d* = 0.78			

The composite latency score was also used to examine the relationship between ToM and decision latency. With age controlled, there was a significant negative correlation between A-ToM-Q social performance and latency, *r* = − 0.25, *p* < 0.001. Further, a one-way ANCOVA comparing social scores for the autistic and non-autistic groups, after controlling for both decision latency and age, confirmed the main effect for group, *F* (1, 166) = 7.64, *p* =  < 0.01, *d* = 0.43, with autistic individuals (adjusted *M* = 4.83, *SE* = 0.15) performing more poorly than non-autistic individuals (adjusted *M* = 5.09, *SE* = 0.18) on the A-ToM-Q social scale, independent of time taken to record their decision.

### Heterogeneity of the ASD Sample

Despite the significant effect of group on A-ToM-Q social performance, the data patterns are not consistent with the position that perspective taking difficulties in autistic individuals are ubiquitous. The distribution of scores on the social and physical sub-scales for the two groups is shown in Table 4. Fewer autistic than non-autistic individuals scored 5 or 6 on the social sub-scale and very low scores (≤ 3) were more prevalent among autistic individuals. As expected, these patterns were not as pronounced on the physical sub-scale.

**Table 4:** Number of participants obtaining each score, and cumulative proportion ≤ each score, on the A-ToM social and physical scales for the autistic and non-autistic samples

Scale & group	Score						
Social	0	1	2	3	4	5	6
Autistic							
Frequency	3	6	5	14	17	25	26
Proportion^Cum^	0.03	0.09	0.15	0.29	0.47	0.73	1.00
Non-autistic							
Frequency	0	0	0	3	8	30	34
Proportion^Cum^	0.00	0.00	0.00	0.04	0.15	0.55	1.00
Physical							
Autistic							
Frequency	0	6	12	27	26	16	9
Proportion^Cum^	0.00	0.06	0.19	0.47	0.74	0.91	1.00
Non-autistic							
Frequency	0	2	4	12	23	23	11
Proportion^Cum^	0.00	0.03	0.08	0.24	0.55	0.85	1.00

The latency data, however, paint a different picture. For economy of presentation, we only show the age-adjusted latency patterns for those scoring 5 or 6 on the social sub-scale (see Table 5). Comparison of the descriptive and inferential statistics in Table 5 with the age-adjusted patterns for the full samples shown in Table 3 reveal that, even when the autistic individuals performed with high accuracy, decision latencies were still significantly longer than those for non-autistic individuals on four of the six scenarios. Note, however, that for two of those four scenarios, the effect sizes were suggestive of relatively weak effects.

**Table 5:** Decision latency descriptive statistics (in sec) and one-way ANCOVA inferential statistics (with age as a covariate) for autistic and non-autistic groups’ correct decisions on A-ToM-Q social and physical items for individuals with social scores of 5 or 6

A-ToM-Q item	Autistic		Non-autistic	
	Adjusted mean	SE	Adjusted mean	SE
Social				
Bunnies	10.36	0.91	7.28	0.80
	*F* (1, 108) = 5.53, *p* = .021, *d* = 0.46			
Party	16.68	1.42	13.75	1.23
	*F* (1, 111) = 2.05, *p* = .155, *d* = 0.27			
Crying man	7.27	0.87	6.31	0.74
	*F* (1, 110) = 0.61, *p* = .438, *d* = 0.14			
Burglar	11.82	1.10	7.85	1.04
	*F* (1, 92) = 5.70, *p* = .019, *d* = 0.50			
Hat	8.74	0.71	6.51	0.62
	*F* (1, 99) = 4.70, *p* = .033, *d* = 0.44			
Spaghetti	10.19	0.93	6.76	0.79
	*F* (1, 97) = 6.83, *p* = .010, *d* = 0.54			

### Self-Awareness

**Fig. 1:**
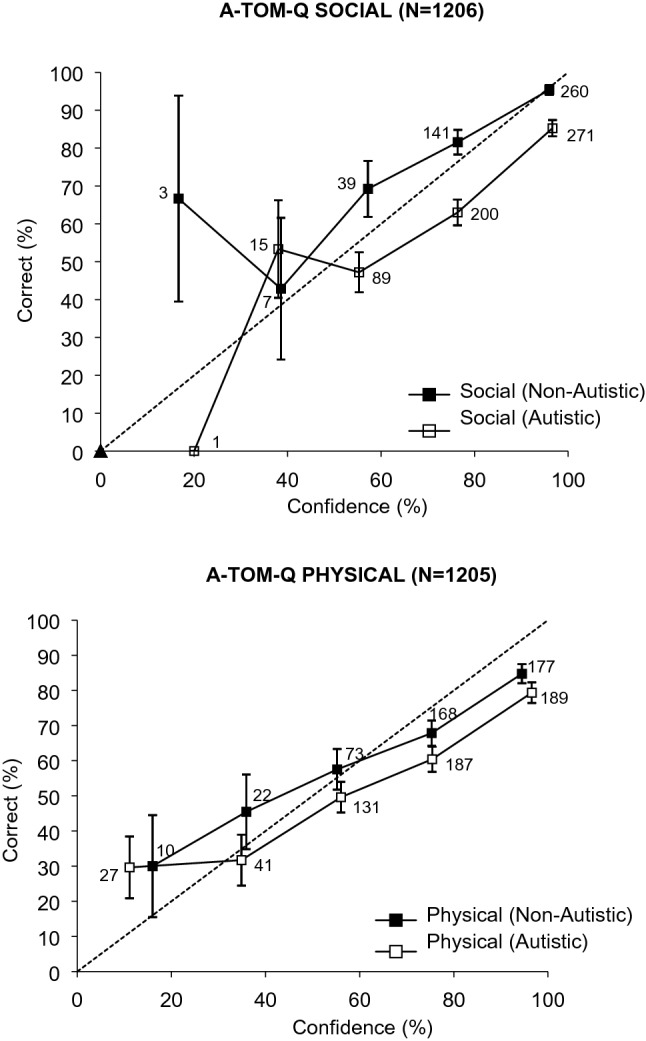
Confidence-accuracy calibration curves for social (upper panel) and physical (lower panel) sub-scales, with number of observations for each plot point

To examine participants’ monitoring of their decision-making accuracy, we plotted decision accuracy against confidence (recorded immediately after the decision) to produce a calibration curve for each group. As participants only completed 6 items on each of the social and physical scales, the calculation of individual calibration statistics was impractical. The calibration curves indicated the proportion of accurate decisions (with every item’s response contributing a separate data point) at each confidence level from 0 to 100%. To maximize the stability of estimates, confidence categories were collapsed into five categories (0–20%, 30–40%, 50–60%, 70–80%, 90–100%), with the proportion correct in each plotted against the weighted mean confidence for that category. Figure 1 shows the calibration curves for the two groups for A-ToM-Q social (upper panel) and physical (lower panel) sub-scales.

For the A-ToM social scale, accuracy for both groups was high at the 90–100% confidence level and declined (following the ideal calibration line) as confidence fell. (The two lowest confidence plot points for each group rely on too few observations to be meaningfully interpreted.) However, although confidence and accuracy were calibrated for both groups, there were clear differences. The non-autistic participants were perfectly calibrated at the highest confidence level and thereafter their curve was characterized by slight under-confidence: that is, proportion correct was a little higher than the associated confidence level. In contrast, the autistic participants’ curve was characterized by overconfidence: for example, only around 80% of decisions made with 90–100% confidence were accurate, only around 60% of decisions made with 70–80% confidence were accurate, and so on. Thus, as well as being less accurate and slower on social items than non-autistic participants, the autistic participants’ confidence judgments did not reflect the same degree of awareness of lapses in accuracy. For the physical items, both groups were characterized by overconfidence in the upper section of the curves (i.e., 70–80% confidence and above).

Interestingly, the curves for autistic individuals on the social scale suggested no meaningful differences between fast and slow decision makers. The sample was split into fast and slow responders based on each individual’s average z-score across items, with separate calibration curves produced for each (see Supplemental Materials Figure S1). Where there were sufficient observations to produce stable estimates (i.e., the curve’s upper section), the curves were almost identical, suggesting similar self-awareness in fast and slow responders. In contrast, slow responders were characterized by overconfidence on the physical scale.

Note, however, that despite the calibration curves providing evidence of greater overconfidence in the autistic participants, they were not more confident overall. A 2 (group: autistic, non-autistic) × 2 (sub-scale: social, physical) mixed ANOVA on median confidence for the 6 items revealed a weak but significant effect of group, *F* (1, 169) = 3.95, *p* < 0.05, *η*_*p*_^*2*^ = 0.02, and a significant effect of sub-scale, *F* (1, 169) = 91.70, *p* < 0.001, *η*_*p*_^*2*^ = 0.35. Autistic individuals were actually less confident (*M* = 83.44, *SD* = 14.06 and *M* = 73.96, *SD* = 17.03 for both social and physical items, respectively) than non-autistic participants (*M* = 86.73, *SD* = 12.91 and *M* = 78.80, *SD* = 13.15, respectively). The lower confidence for autistic participants on both sub-scales is consistent with their significantly longer response latencies.

Finally, for participants from the two groups who scored 5 or 6 on the social scale, there were no meaningful group differences between the social or the physical curves (see Supplemental Materials Figure S2). The social scale curve was characterized by perfect calibration at the highest confidence levels, and considerable underconfidence at lower levels. The physical scale curve indicated overconfidence at the maximum confidence levels, but close to perfect calibration at lower levels.

## Discussion

This study makes two main contributions. First, it indicates the A-ToM-Q is a promising and efficient measure of perspective taking in autistic adults for use with either in-person or online administration. Second, it contributes to our understanding of the nature and extent of perspective taking difficulties that may characterize autistic adults. We address each of these contributions, followed by consideration of limitations and issues for future research.

First, we consider the measure itself. As with the A-ToM (Brewer et al., 2017), performance on the A-ToM-Q demonstrated concurrent validity with other adult ToM measures. Convergent and divergent validity were suggested by meaningful relationships with performance accuracy on measures that are considered to be underpinned by perspective taking ability, and the absence of such relationships with measures that clearly differentiated autistic from non-autistic adults but do not depend on perspective taking ability.

Discriminant validity was indicated by stronger differentiation of autistic and non-autistic samples on social (i.e., perspective taking) than physical scale performance accuracy. Moreover, social, but not physical, performance accuracy was unrelated to verbal ability, and controlling for verbal IQ (using participants who completed the WASI-II) removed the group difference on the physical but not the social sub-scale. In contrast, the two samples were (a) not more strongly differentiated on the social than the physical scale of the Strange Stories test, with those scales correlating significantly with verbal IQ, and (b) not as clearly differentiated on either the Frith- Happé animations social or feelings categorization scales.

From an administration perspective, the A-ToM-Q offers significant advantages over the A-ToM. First, the latency data indicate that the time required to complete the social scale—the scale of interest for researchers seeking a predictive perspective taking measure and for clinicians interested in understanding factors contributing to social-communicative difficulties—will be between 5 and 8 min for a substantial proportion of autistic adults. Scoring, whether manual or electronic, will incur negligible time demands. Moreover, no training in scoring will be required, nor will there be concerns about the reliability of scoring across test administrators. Thus, the potential for web-based administration for clinicians and researchers, together with that for large-scale data collection, is substantial.

The study’s second major contribution is the understanding that different aspects of our data provide regarding the nature and extent of perspective taking difficulties in autistic adults. First, as found with the A-ToM, variability in perspective taking across autistic individuals was substantial. Reiterating Brewer et al.’s (2017) conclusion, a deficit in perspective taking or ToM may suggest autism in adults but is not a requirement to meet diagnostic criteria. This conclusion has implications, of course, for the perspective that a ToM deficit is a core feature of the condition, a topic that more recently has also been taken up by other researchers (e.g., Astle & Fletcher-Watson, 2020; Gernsbacher & Yergeau, 2019).

The second informative feature of our findings is provided by the response latency data. Autistic individuals were significantly slower than non-autistic individuals across the various test items. Controlling for age—with decision latency longer for older participants—reduced but did not eliminate group latency differences. The latency patterns are not consistent with the possibility that poorer perspective taking performance reflected careless or impulsive responding (i.e., sacrificing accuracy for speed of responding). They are, however, consistent with the perspective that autistic individuals are predisposed towards deliberative, slower and more effortful “Type 2” processing, whereas typically developing individuals are predisposed towards a more rapid, effortless and intuitive (“Type 1”) style of decision making (Brosnan et al., 2016b, 2017). Thus, the latency data could reflect a tendency for autistic individuals to analyze meticulously the various response options before responding. Note, however, that doing so did not eliminate the performance differential between the groups, with the significant group difference persisting with the composite latency measure controlled. Our data do not, however, permit any conclusion about whether any apparent predisposition towards deliberate or effortful processing reflects something akin to a ‘fixed’—analogous to an automatic or unconscious cognition—versus an adjustable or strategic (i.e., a conscious or controlled) limitation.

We examined whether further insight into these possibilities might be provided by a comparative examination of those autistic and non-autistic individuals who achieved high levels of accuracy on the social sub-scale. Despite those two sub-samples performing with similar accuracy, the autistic individuals remained slower than the non-autistic individuals, even after controlling for age. Again, these data are consistent with autistic individuals engaging in effortful over intuitive processing. In the Future Research section, we outline a possible approach to clarifying whether such a predisposition is malleable.

The fact the latency differences were relatively consistent across items suggests that consideration of response latencies may provide valuable information for clinicians trying to identify areas of difficulty for clients. Given the relatively small sample sizes for the two groups, the latency data presented in Tables 1 and 3 obviously cannot be regarded as normative data. However, they do provide some potentially useful initial reference points for pinpointing areas of difficulty, even for individuals who respond correctly to an item.

The self-awareness data provide the third contribution to our understanding of perspective taking limitations. Despite being less confident than typically developing individuals when responding correctly—a not unexpected finding if processing is particularly effortful—the confidence-accuracy calibration curves for autistic individuals were characterized by more marked overconfidence than those of non-autistic individuals. Lower accuracy and more marked overconfidence are consistent with what has often been referred to in the broader confidence-accuracy literature as the hard-easy effect, whereby the likelihood of overconfidence increases with task difficulty (e.g., Gigerenzer et al., 1991; Juslin et al., 2000; Weber & Brewer, 2004). Thus, at the group level, autistic individuals not only tended to be less accurate, slower and less confident, but were also less aware of when they were inaccurate. The finding of reduced metacognitive awareness in autistic individuals is consistent with recent research suggesting that the accuracy of metacognitive judgments is dependent on perspective taking ability (Nicholson et al., 2021). In Apperly and Butterfill’s two-system ToM framework, reduced metacognitive awareness would appear to correspond to a less efficient version of the cognitively flexible system which they suggest “enables adults to engage in top-down guidance of social interaction (such as anticipating what the audience of a lecture might know or working out how one misjudged the audience afterward)” (Apperly & Butterfill, 2009, p. 996).

One might speculate, therefore, that lower self-awareness would shape the individual’s processing, perhaps rendering them more hesitant in their decision making. In other words, lower self-awareness would be a mechanism underpinning slower and more deliberative processing. Some aspects of the data are, however, unable to be reconciled with this position. For example, calibration curves characterized by under- rather than overconfidence might have been expected for the autistic group overall. Underconfidence was also observed for those highly accurate individuals from both groups (see Figure S2, top panel), yet the highly accurate autistic individuals were still generally slower than the highly accurate non-autistic individuals. Most telling, however, was the finding that the calibration curves for fast and slow autistic decision makers on the social scale did not differ, suggesting an absence of any relationship between self-awareness and decision latency for perspective taking items.

Indeed, the identical calibration curves for fast and slow autistic decision makers suggest the possibility that slower decision making was not a reflection of task difficulty constraining speed of responding. If slow decision makers required the additional time to make their decision because the task was particularly difficult for them, we might expect the calibration curves to be consistent with a hard-easy effect, with slow individuals characterized by greater overconfidence. We return to a consideration of whether slower decision making might reflect an adjustable (e.g., some kind of strategic) limitation when we consider issues for future research.

The heterogeneity of the autistic sample was also highlighted by the calibration curves for those individuals from both groups who responded very accurately on the social scale. The curves for the two groups were virtually indistinguishable: both groups clearly knew they were accurate when they recorded 90–100% confidence but doubted themselves more than they should have when they expressed confidence in the 50–80% range. One might be inclined to argue that this lack of confidence contributed to the slower responding of autistic individuals, but it does not explain why the non-autistic individuals were faster. Indeed, it is generally considered that response latency, or ease of processing, is one of the important cues driving confidence assessments (e.g., Koriat & Ackerman, 2010; Semmler et al., 2004)—with, for example, participants thinking, “that was easy, I solved it really quickly, I must be right”—rather than the opposite. Perhaps these data, coupled with the finding of lower overall confidence for the autistic individuals, is an indication that their longer latencies reflect a genuine processing difficulty.

Finally, we note that, from a clinical perspective, a very easily obtained confidence assessment for the response to each item can provide an indication of whether the individual is aware of any limitations they might have (e.g., an incorrect response followed by a moderate-high confidence estimate) which might have broader implications for interpretation of self-report measures amongst autistic people.

### Limitations and Future Research

#### Sample Characteristics and Reliability

To secure autistic participant numbers, we collected an online sample to supplement our in-person sample. This raises two issues. First, for the online sub-sample, it meant we were reliant on participants’ self-reports to confirm they had received an ASD diagnosis, a practice that is potentially open to abuse. However, such was our questioning about details of the diagnosis that we believe it is most unlikely that people without a formal diagnosis took part (although the precise assessment procedures used in those diagnoses are, of course, unknown). Moreover, we re-emphasize two aspects of our data that give us some confidence in the integrity of our sample: (1) The markedly higher AQ-10 scores for the online autistic individuals than for the non-autistic sample; and (2) The absence of any significant differences between the online and in-person autistic individuals on the A-ToM-Q sub-scales, the Strange Stories and the Frith-Happé animations.

Second, the inability to administer the WASI-II to online participants meant the verbal ability measures for the two autistic sub-samples differed (i.e., WASI-II VCI vs. AVT). Thus, it is possible that the online sample differed in verbal ability from the non-autistic sample. Confirming that the two samples were of equivalent verbal ability is obviously not possible, despite our exclusion of individuals who scored more than two standard deviations below the normative sample mean on the AVT. Nevertheless, given (a) the negligible correlations between the key measure of interest, the A-ToM-Q social scale, and both verbal ability measures (i.e., the WASI-II’s VCI and the AVT), and (b) performance on the social scale was independent of verbal ability, it seems unlikely that the differences we have reported between the autistic and non-autistic samples were IQ-related. Nevertheless, replication of our findings with a consistent verbal ability measure across groups would be desirable.

We emphasize that our controls for verbal ability do not rule out the possibility that there may be crucial language factors at play that undermine the interpretation of verbal (or written, in the case of the Strange Stories test) communications required in some ToM tasks (see, for example, Astington & Baird, 2005). Sub-tests such as the vocabulary and similarities scales from formal tests of verbal ability clearly do not capture all the subtleties and complexities of either receptive or expressive language skills that may underpin how we interpret social communications from our interaction partners. The precise nature of those factors and how they might have been shaped by the individual’s neuro-developmental history are questions our data do not address.

Although Brewer et al. (2017) reported impressive test–retest reliability (*r* = 0.82) for the A-ToM social scale at intervals ranging from 2 to 83 weeks (*M* = 23.7 weeks), we were unable to collect test–retest data for the online sample. Thus, future work gathering test–retest data on the A-TOM-Q would be desirable, bearing in mind that the intervening interval should be relatively long given the possibility that participants might recall forced-choice responses.

Large sample sizes in studies such as this are desirable, especially to gain an appreciation of subtle variations that may be associated with factors such as gender, age, and degree of autistic traits. (Note, for example, the impact of controlling for age on the decision latency outcomes.) Another reason for large sample replication of our study is suggested by the latency data. The descriptive latency statistics provide useful guidelines for clinicians regarding possible perspective taking difficulties of examinees. Given the inherent inter-individual variability of latency measures, larger samples should ensure greater stability of those guidelines. Useful benchmark latency data could be provided by a large (and easily obtainable) sample of non-autistic individuals, with subsequent identification of abnormally long response latencies from autistic examinees achievable by reference to the distributional characteristics of latencies from a large and diverse sample of non-autistic individuals.

#### Other Issues for Future Research

We also suggest several other areas for future research. First, issues already raised about the response latency data deserve attention if we are to understand fully the mechanisms underpinning perspective taking difficulties and, especially, the heterogeneous nature and extent of those difficulties. Our data are consistent with the position that autistic individuals are less likely than non-autistic individuals to engage in intuitive processing and more likely predisposed towards deliberative or effortful processing (cf. Brosnan et al., 2016b, 2017). What is not clear is whether such a processing mode reflects a fixed constraint on decision latency, or might be controllable or manipulable by some kind of intervention. Brosnan et al.'s conclusions were based on self-reports of processing mode preferences and performance on the Cognitive Reflections Test (CRT; Frederick, 2005). That the adoption of a deliberative processing mode by autistic individuals in the Brosnan et al. studies was not ubiquitous leaves open the possibility that this may be an adjustable characteristic. Indeed, Evans and Curtis-Holmes (2005) showed that, for non-autistic individuals at least, it was possible to manipulate the processing mode employed by requiring rapid responses on a reasoning task. How constraining deliberative processing affects performance on all decision-making tasks is an empirical question—and how autistic individuals are affected when deliberative processing is constrained on a perspective taking task also awaits empirical investigation.

One approach to clarifying this issue would be to measure A-ToM-Q social performance using a response-signal deadline procedure (cf. Brewer & Smith, 1990; Pachella & Fisher, 1969). With a deadline procedure, participants are required to respond to the stimulus prior to a deadline. By varying the deadline across trials or trial blocks (e.g., 1 s, 2 s, 3 s, 4 s, 5 s) a range of fast responses is produced, allowing the relationship between processing time and response accuracy to be determined empirically. For example, if under a deadline procedure both autistic and non-autistic participants achieved asymptotic and equivalent accuracy at a 3 s deadline, but their average response latencies under non-deadline conditions were 12 s and 5 s, respectively, the slower responding of the former group clearly does not reflect a processing capacity limitation. If, however, the performance accuracy differential persisted at shorter deadlines, a more fundamental and non-adjustable limitation is likely. We also note that, although deadline responding may reduce the accuracy differential, it might cause some discomfort for autistic individuals if incompatible with their preferred approach to a task. Thus, it would be interesting to measure participants’ affective reactions when responding under deadline conditions, particularly given the practical implications this has for real-life contexts, ranging from everyday social interactions to employment interviews.

Second, we note that although some research has examined the A-ToM’s criterion-related validity (e.g., Young & Brewer, 2020), new research along similar lines would be extremely valuable if we are to unravel the relationships between individuals’ daily functioning and the nature and extent of perspective taking, or ToM, difficulties. The capacity of the A-ToM-Q to supplement accuracy measures with latency and confidence measures offers potential for pinpointing specific processing difficulties, as well as problems at the metacognitive level that may constrain adaptive responding and future learning. Just like the student who is blissfully unaware of their inadequate knowledge leading up to a test and, hence, does not study appropriately, so the individual who does not appreciate that they are not ‘reading’ some of the subtle signals from their interaction partners is not only less likely to respond in a socially appropriate manner during interpersonal interactions, but is also likely to be ignorant of, and unlikely to explore, potentially beneficial avenues for social development.

Third, we are mindful of the caveat previously expressed about the range of perspective taking behaviors tapped by the A-ToM (see, for example, Brewer et al., 2017). The behaviors sampled by the A-ToM and, in turn, the A-ToM-Q, were based on an item analysis of a larger array of test items. However, it is important to bear in mind that it is unlikely that the item set captures the range or indeed the extent of perspective taking difficulties that some autistic individuals may experience. In other words, there is likely considerable scope for ongoing research into the nature and measurement of such difficulties.

Fourth, like the A-ToM, the A-ToM-Q provides participants with explicit prompts to reflect on the behaviors observed in the videos, with the multiple-choice options providing additional structure for those reflections. Given previous demonstrations that autistic individuals may not spontaneously display ToM in the absence of specific prompts (e.g., Senju et al., 2009), it will be important to examine whether our measure, like various other measures of ToM, may overestimate the ability of some autistic individuals to apply ToM in real life tasks that will often lack such prompts. For example, how might autistic and non-autistic individuals’ performance compare with a very general instruction such as “Please tell us what was going on in that scenario?”.

In sum, the A-ToM-Q’s social scale provides a tool to assess perspective taking difficulties far more rapidly than is possible with its predecessor, the A-ToM, and to obtain a more comprehensive picture by means of the access to latency and self-awareness data. The availability of such measures offers benefits to researchers interested in elucidating the nature of the difficulties some autistic individuals may experience, as well as the potential for large-scale data collection because of its compatibility with web-based administration. From a clinical perspective, the test stimuli depict realistic social interactions that are likely to be valuable for clinicians seeking to provide clients with specific examples that highlight the nuanced nature of interpersonal interactions. In such contexts, responses that meet the “partially correct” criteria (see Supplemental Materials Table S1) may be informative for clinicians trying to pinpoint the nature of any difficulties that clients are experiencing.

There are other obvious issues for future research. With respect to this particular measurement instrument it will be important to clarify issues such as the generality of particular difficulties highlighted by A-ToM-Q performance. For example, are those difficulties alleviated in any way when there is a greater degree of contextual information available than is provided in the instrument’s test items, and will the heterogeneity of performance witnessed in participants in this study characterize individuals’ behavior across a broad range of perspective taking scenarios?

A broader issue is the continued investigation of the optimal approaches for developing flexible and sustainable perspective taking skills in individuals who are experiencing difficulties. Although resolving this question is beyond the scope of this study—for detailed overviews see, for example, Brewer and Young (2015) and Fletcher-Watson et al. (2014)—we very briefly highlight just some of the considerations involved. For example, should the focus be on trying to improve specific areas or dimensions of perspective taking or some broad or pervasive social-cognitive capacity? At what stage(s) of the individual’s development should the efforts be focused in order to maximize any benefits? How much and precisely what form of intervention will be necessary to promote maintenance of newly acquired skills and what special steps will be needed to ensure generalization or transfer of skills? It is also important to emphasize that, given well-developed ToM underpins much of our social interaction, being able to identify difficulties and subsequently ameliorate them can potentially benefit a wider range of autistic individuals than we sampled in this study. For example, benefits for some non-verbal autistic individuals could be realized if future work can adapt instruments like the A-ToM-Q for use with such individuals. These are just some of the critical questions that researchers and clinicians will need to tackle—but first, of course, we need to be able to identify the nature and extent of any difficulties.

### Supplementary Information

Below is the link to the electronic supplementary material.

Supplementary file1 (DOCX 241 kb)

## Example Scripts and Response Options for Social Items

### *A-ToM Item*: Bunnies*Item Type*: Persuasion

Two women sit in their living room discussing their bunnies:

SUSIE: “So you know there is a lady coming over today to take a look at the rabbits.”

MRS SMITH: “That’s good, because you know we can’t keep them all.”

SUSIE: “I know.”

She looks sad as she picks up one of the bunnies and cuddles it.

SUSIE: “I just love them so much. I can’t bear the thought of anything bad happening to them. They’re just so beautiful and cuddly.”

A girl approaches the house and knocks on the front door. The door opens to reveal woman 1 and woman 2:

POTENTIAL BUYER: “Hi, I’m here to look at the bunnies.”

SUSIE: “Of course, come inside.”

Mrs Smith, Susie and the potential buyer are sitting in the living room. The potential buyer is cuddling one of the bunnies.

POTENTIAL BUYER: “Oh they are all so cute. It’s a shame they’re all have males though, I was really looking for a female bunny.”

SUSIE: “Oh that is a shame. You know if I can’t find a good home for them, I’m going to have to drown them.”

Question to Participant: Why does she say she will have to drown the rabbits?

Please answer each of the following questions as quickly and accurately as you possibly can.

Why does she say she will have to drown the rabbits?

- She is trying to make the person feel guilty so they will buy one of the rabbits. (correct)
- She is trying to get the girl to buy one. (partially correct)
- She is unable to keep them all and if she can’t she will have to kill them. (incorrect)
- She’s a horrible person who hates rabbits. (incorrect)

*******************************

### *A-ToM Item*: Party*Item Type*: Faux Pas

Simon and Dave are standing in the corner of a party:

SIMON: “So my brother knows the guy who owns this place.”

DAVE: “That’s funny, my brother is the guy who owns this place.”

They laugh together.

SIMON: “Nice. I know this might be a bit forward, but I was wondering if I could grab your number?”.

DAVE: “Sure, but if you don’t mind, can you not tell anyone about it, as my father doesn’t know I’m gay. Only my brother knows.”

SIMON: “Yeah that’s cool, I know it’s hard. My family knows but they seem pretty chill with it.”

On the other side of the room Rob and Pete, are chatting to Dave’s Dad:

ROB: “So, Mr Jones it looks like my brother and your son are really hitting it off. They make a cute couple.”

PETE (trying to cover it up): “Ah… Rob did you watch that footy game last night?”.

Dave’s dad ignores what Pete says:

DAD (To Rob): “Sorry, ‘hitting it off’? What are you implying?”.

ROB (Realising what he has said) “Uh, nothing.”

He turns and faces Pete:

ROB: “Yeah, I saw the game! It was epic.”

Question to Participant: Was there anything awkward or uncomfortable in this interaction? If so, what was it?

- Yes, the American has now let the father in on the secret that his brother and the man’s son are gay and the father clearly didn’t know. (correct)
- Yes. (partially correct)
- No, the American who knew his brother was gay was telling the father of the other boy that they made a cute couple. (incorrect)
- No, the conversation appears quite reasonable and no-one appears uncomfortable. (incorrect)

## Footnotes

1 For both groups, correlations between age and A-ToM-Q performance were negligible on both the social, *r* = .07 and -.10 for autistic and non-autistic samples respectively, and physical sub-scales, *r* = .04 and -.08 for autistic and non-autistic samples respectively. Note also that Pearson *r* coefficients are reported throughout; however, the pattern and magnitude of non-parametric coefficients are very similar.

## References

[CR1] Abell F, Happé F, Frith U (2000). Do triangles play tricks? Attribution of mental states to animated shapes in normal and abnormal development. Cognitive Development.

[CR2] Allison C, Auyeung B, Baron-Cohen S (2012). Toward brief “red flags” for autism screening: The short autism spectrum quotient and the short quantitative checklist in 1,000 cases and 3,000 controls. Journal of the American Academy of Child and Adolescent Psychiatry.

[CR03] American Psychiatric Association. (2000). *Diagnostic and statistical manual of mental disorders* (4th ed., text rev.). Washington, DC: American Psychiatric Publishing

[CR04] American Psychiatric Association. (2013). *Diagnostic and statistical manual of mental disorders* (5th ed.). Washington, DC: American Psychiatric Publishing

[CR3] Apperly IA, Butterfill SA (2009). Do humans have two systems to track beliefs and belief-like states?. Psychological Review.

[CR4] Astington J, Baird JA (2005). Why language matters for theory of mind.

[CR5] Astle DE, Fletcher-Watson S (2020). Beyond the core-deficit hypothesis in developmental disorders. Current Directions in Psychological Science.

[CR6] Baranski JV, Petrusic WM (1994). The calibration and resolution of confidence in perceptual judgments. Perception and Psychophysics.

[CR7] Baron-Cohen S (1995). Mindblindness: An essay on autism and theory of mind.

[CR8] Baron-Cohen S, Leslie AM, Frith U (1985). Does the autistic child have a “theory of mind”?. Cognition.

[CR9] Baron-Cohen S, Wheelwright S, Hill J, Raste Y, Plumb I (2001). The “Reading the Mind in the Eyes” test revised version: A study with normal adults, and adults with Asperger syndrome or high-functioning autism. Journal of Child Psychology and Psychiatry.

[CR10] Baron-Cohen S, Wheelwright S, Skinner R, Martin J, Clubley E (2001). The autism spectrum quotient (AQ): Evidence from Asperger syndrome/high-functioning autism, males and females, scientists and mathematicians. Journal of Autism and Developmental Disorders.

[CR11] Brewer N, Smith GA (1984). How normal and retarded individuals monitor and regulate speed and accuracy of responding in serial choice tasks. Journal of Experimental Psychology: General.

[CR12] Brewer N, Smith GA (1989). Developmental changes in processing speed: Influence of speed-accuracy regulation. Journal of Experimental Psychology: General.

[CR13] Brewer N, Smith GA (1990). Processing speed and mental retardation: Deadline procedures indicate fixed and adjustable limitations. Memory and Cognition.

[CR14] Brewer N, Wells GL (2006). The confidence-accuracy relationship in eyewitness identification: Effects of lineup instructions, foil similarity and target-absent base rates. Journal of Experimental Psychology: Applied.

[CR15] Brewer N, Young RL (2015). Crime and autism spectrum disorder: Myths and mechanisms.

[CR16] Brewer N, Young RL, Barnett E (2017). Measuring theory of mind in adults with autism spectrum disorder. Journal of Autism and Developmental Disorders.

[CR17] Brewer N, Zoanetti J, Young RL (2019). Convergent validity of the A-ToM (Adult Theory of Mind) test for individuals with autism spectrum disorder. Journal of Psychoeducational Assessment.

[CR18] Brosnan M, Johnson H, Grawemeyer B, Chapman E, Antoniadou K, Hollinworth M (2016). Deficits in metacognitive monitoring in mathematics assessments in learners with autism spectrum disorder. Autism.

[CR19] Brosnan M, Lewton M, Ashwin C (2016). Reasoning on the autism spectrum: A dual process theory account. Journal of Autism and Developmental Disorders.

[CR20] Brosnan M, Ashwin C, Lewton M (2017). Brief report: Intuitive and reflective reasoning in autism spectrum disorder. Journal of Autism and Developmental Disorders.

[CR21] Connor KM, Kobak KA, Churchill LE, Katzelnick D, Davidson JR (2001). Mini-SPIN: A brief screening assessment for generalized social anxiety disorder. Depression and Anxiety.

[CR22] Critchley HD, Daly EM, Bullmore ET, Williams SCR, Van Amelsvoort T, Robertson DM, Rowe A, Phillips M, McAlonan G, Howlin P, Murphy DGM (2000). The functional neural anatomy of social behaviour: Changes in cerebral blood flow when people with autistic disorder process facial expressions. Brain.

[CR23] Davis MH (1983). Measuring individual differences in empathy: Evidence for a multidimensional approach. Journal of Personality and Social Psychology.

[CR24] Dvash J, Shamay-Tsoory SG (2014). Theory of mind and empathy as multidimensional constructs: Neurological foundations. Topics in Language Disorders.

[CR25] Dziobek I, Fleck S, Kalbe E, Rogers K, Hassesnstab J, Brand M, Kessler J, Woike JK, Wolf OT, Convit A (2006). Introducing MASC: A movie for the assessment of social cognition. Journal of Autism and Developmental Disorders.

[CR26] Ekstrom RB, French JW, Harman HH, Dermen D (1976). Kit of factor-referenced cognitive tests.

[CR27] Evans JStBT, Curtis-Holmes J (2005). Rapid responding increases belief bias: Evidence for the dual-process theory of reasoning. Thinking and Reasoning.

[CR28] Fletcher-Watson S, McConnell F, Manola E, McConachie H (2014). Intervention based on the theory of mind cognitive model for Autism Spectrum Disorder (ASD).

[CR29] Frederick S (2005). Cognitive reflection and decision making. Journal of Economic Perspectives.

[CR30] Gernsbacher MA, Yergeau M (2019). Empirical failures of the claim that autistic people lack a theory of mind. Archives of Scientific Psychology.

[CR31] Gigerenzer G, Hoffrage U, Kleinbölting H (1991). Probabilistic mental models: A Brunswikian theory of confidence. Psychological Review.

[CR32] Globerson E, Amir N, Kishon-Rabin L, Golan O (2015). Prosody recognition in adults with high-functioning autism spectrum disorders: From psychoacoustics to cognition. Autism Research.

[CR33] Grainger C, Williams DM, Lind SE (2016). Metacognitive monitoring and control processes in children with autism spectrum disorder: Diminished judgement of confidence accuracy. Consciousness and Cognition: An International Journal.

[CR34] Happé, F. (1999). *Instructions for theory of mind story task*. Unpublished document provided by F. Happé

[CR35] Heavey L, Phillips W, Baron-Cohen S, Rutter R (2000). The awkward moments test: A naturalistic measure of social understanding in autism. Journal of Autism and Developmental Disorders.

[CR36] Juslin P, Olsson N, Winman A (1996). Calibration and diagnosticity of confidence in eyewitness identification: Comments on what can be inferred from the low confidence-accuracy correlation. Journal of Experimental Psychology: Learning, Memory, and Cognition.

[CR37] Juslin P, Winman A, Olsson H (2000). Naive empiricism and dogmatism in confidence research: A critical examination of the hard-easy effect. Psychological Review.

[CR38] Kelly KJ, Metcalfe J (2011). Metacognition of emotional face recognition. Emotion.

[CR39] Koriat A, Ackerman R (2010). Choice latency as a cue for children’s subjective confidence in the correctness of their answers. Developmental Science.

[CR40] Livingston LA, Carr B, Shah P (2019). Recent advances and new directions in measuring theory of mind in autistic adults. Journal of Autism and Developmental Disorders.

[CR42] McNeish D (2018). Thanks coefficient alpha, we’ll take it from here. Psychological Methods.

[CR43] Maras K, Norris J, Brewer N (2020). Metacognitive monitoring and control of eyewitness memory reports in autism. Autism Research.

[CR044] Miu, A. C., Pană, S. E., & Avram, J. (2012). Emotional face processing in neurotypicals with autistic traits Pană, Julia Avram: Implications for the broad autism phenotype. *Psychiatry Research*, *198*, 489-494. 10.1016/j.psychres.2012.01.02 10.1016/j.psychres.2012.01.024 22425467

[CR44] Nicholson T, Williams DM, Lind SE, Grainger C, Carruthers P (2021). Linking metacognition and mindreading: Evidence from autism and dual task investigations. Journal of Experimental Psychology: General.

[CR45] Nuske HJ, Vivanti G, Dissanayake C (2013). Are emotion impairments unique to, universal, or specific in autism spectrum disorder?. A Comprehensive Review. Cognition and Emotion.

[CR46] Pachella RC, Fisher DF (1969). Effect of stimulus degradation and similarity on the trade-off between speed and accuracy in absolute judgments. Journal of Experimental Psychology.

[CR47] Sawyer ACP, Williamson P, Young R (2014). Metacognitive processes in emotion recognition: Are they different in adults with Asperger’s disorder?. Journal of Autism and Developmental Disorders.

[CR48] Semmler C, Brewer N, Wells GL (2004). Effects of postidentification feedback on eyewitness identification and nonidentification confidence. Journal of Applied Psychology.

[CR49] Senju A, Southgate V, White S, Frith U (2009). Mindblind eyes: An absence of spontaneous theory of mind in Asperger syndrome. Science.

[CR50] Smith GA, Brewer N (1995). Slowness and age: Speed-accuracy mechanisms. Psychology and Aging.

[CR51] Stone VE, Gerrans P (2006). What’s domain-specific about theory of mind?. Social Neuroscience.

[CR52] Van de Cruys S, Evers K, Van der Hallen R, Van Eylen L, Boets B, de Wit L, Wagemans J (2014). Precise minds in uncertain worlds: Predictive coding in autism. Psychological Review.

[CR53] Weber N, Brewer N (2004). Confidence-accuracy calibration in absolute and relative face recognition judgments. Journal of Experimental Psychology: Applied.

[CR54] Wechsler D (2011). Wechsler Abbreviated Scale of Intelligence (WASI-II).

[CR55] White SJ, Coniston D, Rogers R, Frith U (2011). Developing the Frith-Happé animations: A quick and objective test of theory of mind for adults with autism. Autism Research.

[CR56] Young RL, Brewer N (2020). Brief report: Perspective taking deficits, autism spectrum disorder, and allaying police officers’ suspicions about criminal involvement. Journal of Autism and Developmental Disorders.

