# Supplementary material for: A Quick Measure of Theory of Mind in Autistic Adults: Decision Accuracy, Latency and Self-Awareness
Source: J Autism Dev Disord. 2021 Jun 28;52(6):2479–96. doi: 10.1007/s10803-021-05166-7 (PMC9114060; doi:10.1007/s10803-021-05166-7)
Supplement: Supplementary file 1 — Supplementary file1 (DOCX 241 kb) [file 10803_2021_5166_MOESM1_ESM.docx]

**Supplemental Materials**

**The A-ToM-Q: A quick form of the Adult Theory of Mind (A-ToM) test**

**Table S1.**

**A-ToM-Q Forced-Choice Items**

**Correct answer: green** (only these answers received 1 point in this study)

**Partially correct: blue** (0 points)

**Incorrect: red** (0 points)

Note: The four forced-choice responses were randomly ordered, not as displayed below.

**Please answer each of the following questions as quickly and accurately as you possibly can.**

**SOCIAL ITEMS**

***Why does she say she will have to drown the rabbits?***

1. She is trying to make the person feel guilty so they will buy one of the rabbits.
2. She is trying to get the girl to buy one.
3. She is unable to keep them all and if she can’t she will have to kill them.
4. She’s a horrible person who hates rabbits.

***Was there anything awkward or uncomfortable in this interaction? If so what was it?***

1. Yes, the American has now let the father in on the secret that his brother and the man’s son are gay and the father clearly didn’t know.
2. Yes.
3. No, the American who knew his brother was gay was telling the father of the other boy that they made a cute couple.
4. No, the conversation appears quite reasonable and no-one appears uncomfortable.

***Why did the lady say "he is doing just fine”?***

1. The man is clearly not fine. She is being sarcastic.
2. The man is not fine but you don’t know why she said this.
3. He may not be ok, but the woman has misunderstood.
4. The woman is not interested in the man.

***Why does the burglar give himself up?***

1. The burglar thought the policeman was calling out because he knew of the burglar’s wrong doing.
2. The burglar thought he was being arrested.
3. The burglar had a guilty conscience.
4. The policeman had his glove.

***Why does she say she loves the hat?***

1. She told a white lie to spare the aunt’s feelings.
2. She didn’t want to be rude.
3. She thought it was the right thing to do.
4. She really did like the hat.

***When the mother said ‘that meal must have filled you up’ did she mean it? If not, why did she say it?***

1. No, she was being sarcastic.
2. Yes, the boy must have been quite full.
3. No, but there is no reason for her to say this.
4. No, the boy didn’t eat much.

**PHYSICAL ITEMS**

***Why does he buy the pack of 10?***

1. It was more economical.
2. It was good to have some spare light bulbs.
3. He will need more later.
4. He likes that brand the best.

***Why did Harry win?***

1. Harry is better in the ocean.
2. Harry is generally a better swimmer.
3. Harry is older.
4. Harry is male.

***Why is the post office the most likely place to look?***

1. The post office was the last place where she was likely to use her glasses.
2. The post office was where she left them.
3. It was the last place she had been.
4. The post office was the closest place to them.

***Why does he accept the dealer’s offer to pay in monthly instalments?***

1. Because his bank pays better interest than the dealer charges.
2. He could save money.
3. He thought it was the sensible thing to do.
4. He can’t afford the whole thing.

***Why does she need an x-ray?***

1. There may be an injury to her leg and there is a need to see if she has broken anything.
2. Because her leg is swollen.
3. To see what’s wrong.
4. It’s standard procedure.

***Why did Mrs Simpson put the book in a special room?***

1. The book is delicate and requires special handling to preserve it.
2. The book is old.
3. It is a special book.
4. So she would always know where to find it.

Table S2

*Inter-correlations of A-ToM-Q scales with concurrent ToM measures*

| Measure | 1. | 2. | 3. | 4. | 5. | 6. |
| --- | --- | --- | --- | --- | --- | --- |
| 1. A-ToM-Q social | - | .39** | .45** | .40** | .17* | .28** |
| 2. A-ToM-Q physical |  | - | .36** | .37** | .10 | .22** |
| 3. Strange Stories social |  |  | - | .61** | .09 | .31** |
| 4. Strange Stories physical |  |  |  | - | .17* | .36** |
| 5. Frith-Happé mental |  |  |  |  | - | .79** |
| 6. Frith-Happé feelings |  |  |  |  |  | - |

**p* < .05, ***p* < .01 (2-tailed)

Table S3

*Descriptive statistics for the A-ToM-Q and the various concurrent, convergent and divergent validity measures for the autistic and non-autistic samples*.

|  | Group | |
| --- | --- | --- |
| Scale | Autistic  (*N* = 96) | Non-autistic  (*N* = 75) |
| A-ToM-Q Social |  |  |
| Mean | 4.24 | 5.27 |
| Standard deviation | 1.65 | 0.81 |
| 95% CIs | 3.91 – 4.57 | 5.09 – 5.45 |
| Cohen’s *d* | 0.76 | |
| 95% CIs around *d* | 0.45 – 1.07 | |
| A-ToM-Q Physical |  |  |
| Mean | 3.64 | 4.25 |
| Standard deviation | 1.33 | 1.21 |
| 95% CIs | 3.37 – 3.91 | 3.98 – 4.52 |
| Cohen’s *d* | 0.47 | |
| 95% CIs around *d* | 0.16 – 0.78 | |
| Concurrent Measures |  |  |
| Strange Stories Social |  |  |
| Mean | 12.77 | 14.31 |
| Standard deviation | 3.03 | 1.64 |
| 95% CIs | 12.16 – 13.38 | 13.93 – 14.69 |
| Cohen’s *d* | 0.61 | |
| 95% CIs around *d* | 0.30 – 0.92 | |
| Inferential contrast | *t* (148.57) = 4.23, *p* < .001 | |
| Strange Stories Physical |  |  |
| Mean | 12.01 | 13.99 |
| Standard deviation | 2.82 | 1.70 |
| 95% CIs | 11.44 – 12.58 | 13.60 – 14.38 |
| Cohen’s *d* | 0.83 | |
| 95% CIs around *d* | 0.51 – 1.14 | |
| Inferential contrast | *t* (158.14) = 5.65, *p* < .001 | |
| Frith-Happé animations |  |  |
| Random |  |  |
| Mean | 3.50 | 3.26 |
| Standard deviation | 0.89 | 0.98 |
| 95% CIs | 3.32 – 3.68 | 3.03 – 3.49 |
| Cohen’s *d* | 0.26 | |
| 95% CIs around *d* | -0.04 – 0.56 | |
| Inferential contrast | *t* (168) = 1.69, *p* = .09 | |
| Goal directed |  |  |
| Mean | 2.59 | 2.61 |
| Standard deviation | 1.06 | 1.0 |
| 95% CIs | 2.38 – 2.80 | 2.38 – 2.84 |
| Cohen’s *d* | 0.02 | |
| 95% CIs around *d* | -0.28 – 0.32 | |
| Inferential contrast | *t* (168) = 0.09, *p* = .93 | |
| Mental |  |  |
| Mean | 2.53 | 2.96 |
| Standard deviation | 1.33 | 1.14 |
| 95% CIs | 2.26 – 2.80 | 2.70 – 3.32 |
| Cohen’s *d* | 0.34 | |
| 95% CIs around *d* | 0.03 – 0.64 | |
| Inferential contrast | *t* (166.05) = 2.26, *p* < .05 | |
| Feelings Categorization |  |  |
| Mean | 3.11 | 4.08 |
| Standard deviation | 2.31 | 2.10 |
| 95% CIs | 2.65 – 3.57 | 3.59 – 4.57 |
| Cohen’s *d* | 0.44 | |
| 95% CIs around *d* | 0.13 – 0.75 | |
| Inferential contrast | *t* (168) = 2.82, *p* = < .01 | |
| Convergent Measures |  |  |
| Social Behavioral Skills |  |  |
| Mean | 3.69 | 3.97 |
| Standard deviation | 0.40 | 0.35 |
| 95% CIs | 3.59 – 3.79 | 3.89 – 4.05 |
| Cohen’s *d* | 0.75 | |
| 95% CIs around *d* | 0.41 – 1.09 | |
| Inferential contrast | *t* (138) = 4.42, *p* < 001 | |
| Interpersonal Relations |  |  |
| Mean | 3.80 | 4.32 |
| Standard deviation | 0.64 | 0.44 |
| 95% CIs | 3.64 – 3.96 | 4.21 – 4.43 |
| Cohen’s *d* | 0.97 | |
| 95% CIs around *d* | 0.62 – 1.32 | |
| Inferential contrast | *t* (111.88) = 5.56, *p* < 001 | |
| IRI Perspective Taking |  |  |
| Mean | 12.89 | 18.51 |
| Standard deviation | 6.02 | 3.91 |
| 95% CIs | 11.67 – 14.11 | 17.61 – 19.41 |
| Cohen’s *d* | 1.08 | |
| 95% CIs around *d* | 0.76 – 1.41 | |
| Inferential contrast | *t* (163.93) = 7.37, *p* < 001 | |
| IRI Empathic Concern |  |  |
| Mean | 17.26 | 17.35 |
| Standard deviation | 5.59 | 2.88 |
| 95% CIs | 16.14 – 18.38 | 16.70 – 18.00 |
| Cohen’s *d* | 0.02 | |
| 95% CIs around *d* | -0.28 – 0.32 | |
| Inferential contrast | *t* (147.36) = 0.13, *p* = .90 | |
| IRI Personal Distress |  |  |
| Mean | 14.83 | 12.36 |
| Standard deviation | 5.24 | 4.63 |
| 95% CIs | 11.67 – 14.11 | 11.29 – 13.43 |
| Cohen’s *d* | 0.50 | |
| 95% CIs around *d* | 0.19 – 0.81 | |
| Inferential contrast | *t* (169) = 3.22, *p* < .01 | |
| Mini-SPIN |  |  |
| Mean | 7.70 | 5.81 |
| Standard deviation | 3.15 | 2.51 |
| 95% CIs | 7.06 – 8.34 | 5.23 – 6.39 |
| Cohen’s *d* | 0.65 | |
| 95% CIs around *d* | 0.34 – 0.96 | |
| Inferential contrast | *t* (168.91) = 4.35, *p* < .001 | |

Table S4

*Inter-correlations (r_s_) of A-ToM-Q individual social item latency z-scores*

| Measure | 1. | 2. | 3. | 4. | 5. | 6. |
| --- | --- | --- | --- | --- | --- | --- |
| 1. Bunnies | - | .46** | .50** | .47** | .49** | .40** |
| 2. Party |  | - | .49** | .47** | .44** | .37** |
| 3. Crying man |  |  | - | .40** | .48** | .43** |
| 4. Burglar |  |  |  | - | .41** | .41** |
| 5. Hat |  |  |  |  | - | .29** |
| 6. Spaghetti |  |  |  |  |  | - |

**p* < .05, ***p* < .01 (2-tailed)

*Inter-correlations (r_s_) of A-ToM-Q individual physical item latency z-scores*

| Measure | 1. | 2. | 3. | 4. | 5. | 6. |
| --- | --- | --- | --- | --- | --- | --- |
| 1. Lightbulb | - | .33^**^ | .30^**^ | .32^**^ | .34^**^ | .35^**^ |
| 2. Swimming |  | - | .24^**^ | .40^**^ | .40^**^ | .37^**^ |
| 3. Glasses |  |  | - | .29^**^ | .25^**^ | .32^**^ |
| 4. Car |  |  |  | - | .33^**^ | .43^**^ |
| 5. Leg injury |  |  |  |  | - | .45** |
| 6. Librarian |  |  |  |  |  | - |

**p* < .05, ***p* < .01 (2-tailed)

Figure S1. Confidence-accuracy calibration curves for social (upper panel) and physical (lower panel) sub-scales for fast (filled square) and slow (unfilled square) autistic decision makers, with number observations for each plot point.

**A-ToM-Q SOCIAL, AUTISTIC (N=576)**

**A-ToM-Q PHYSICAL, AUTISTIC (N=575)**

Figure S2. Confidence-accuracy calibration curves for social (upper panel) and physical (lower panel) sub-scales for individuals scoring 5 or 6 on the social sub-scale, with number observations for each plot point.

**A-TOM-Q SOCIAL (Scorers of 5 and 6 only, N=690)**

Social (Non-Autistic)
Social (Autistic)

**A-TOM-Q PHYSICAL (Scorers of 5 and 6 only, N=690)**

Physical (Non-Autistic)
Physical (Autistic)
